# “At our age, we would like to do things the way we want:” a qualitative study of adolescent HIV testing services in Kenya

**DOI:** 10.1097/QAD.0000000000001513

**Published:** 2017-07-01

**Authors:** Kate S. Wilson, Kristin M. Beima-Sofie, Helen Moraa, Anjuli D. Wagner, Cyrus Mugo, Peter M. Mutiti, Dalton Wamalwa, David Bukusi, Grace C. John-Stewart, Jennifer A. Slyker, Pamela K. Kohler, Gabrielle O’Malley

**Affiliations:** a Department of Global Health, University of Washington, Seattle, Washington, USA; b Department of Paediatrics and Child Health, University of Nairobi; c VCT and HIV Prevention Unit/Youth Centre, Kenyatta National Hospital, Nairobi, Kenya; d Department of Medicine; e Department of Epidemiology; f Department of Pediatrics; g Department of Psychosocial and Community Health, University of Washington, Seattle, Washington, USA.

**Keywords:** adolescent HIV, HIV prevention, HIV testing services, linkage to care, social support

## Abstract

**Objectives:**

Adolescents in Africa have low HIV testing rates. Better understanding of adolescent, provider, and caregiver experiences in high-burden countries such as Kenya could improve adolescent HIV testing programs.

**Design:**

We conducted 16 qualitative interviews with HIV-positive and HIV-negative adolescents (13–18 years) and six focus group discussions with Healthcare workers (HCWs) and caregivers of adolescents in Nairobi, Kenya.

**Methods:**

Semi-structured interviews and focus groups were recorded and transcribed. Analysis employed a modified constant comparative approach to triangulate findings and identify themes influencing testing experiences and practices.

**Results:**

All groups identified that supportive interactions during testing were essential to the adolescent's positive testing experience. HCWs were a primary source of support during testing. HCWs who acted respectful and informed helped adolescents accept results, link to care, or return for repeat testing, whereas HCWs who acted dismissive or judgmental discouraged adolescent testing. Caregivers universally supported adolescent testing, including testing with the adolescent to demonstrate support. Caregivers relied on HCWs to inform and encourage adolescents. Although peers played less significant roles during testing, all groups agreed that school-based outreach could increase peer demand and counteract stigma. All groups recognized tensions around adolescent autonomy in the absence of clear consent guidelines. Adolescents valued support people during testing but wanted autonomy over testing and disclosure decisions. HCWs felt pressured to defer consent to caregivers. Caregivers wanted to know results regardless of adolescents’ wishes.

**Conclusion:**

Findings indicate that strengthening HCW, caregiver, and peer capacities to support adolescents while respecting their autonomy may facilitate attaining ‘90-90-90’ targets for adolescents.

## Abstract 2

Supplemental Digital Content is available in the text

## Introduction

HIV testing is the first step in linking HIV-positive adolescents to care and preventing new infections [1,2]. However, the majority of adolescents are unaware of their HIV status [3]. In Kenya, which represents 7% of all adolescent HIV infections globally, 49% of adolescents aged 15–19 years report ever testing for HIV, which is well below the target of 90% [4]. Low uptake of HIV testing represents a missed opportunity for linkage to HIV care and prevention [3,5]. Achieving new United Nations targets to ‘End Adolescent AIDS’ by 2020 [6] will require improved understanding of how to engage adolescents in HIV testing and follow-up services.

Recent studies have documented several individual, social, and systems-level barriers and facilitators to HIV testing and linkage to care among adolescents in Africa [7–13]. Adolescents fear HIV stigma, have inaccurate risk perception, and distrust HCWs [11]. Adolescent uncertainty around how they might have acquired HIV, either sexually or perinatally, can strain relationships with family and sexual partners, further deterring testing. In contrast, desire to know status, prior testing experience, discussing testing with family, and availability of ‘youth friendly services’ (e.g., flexible hours and separate waiting areas) may facilitate testing [10,14,15]. Among HIV-positive adolescents, service quality, coordination between testing and care services, and psychosocial support may increase linkage to care [12,16–18]. Poor coping after a positive result and nondisclosure of status to parents and peers may negatively impact linkage [11,19,20].

Despite growing awareness of these barriers and facilitators to HIV testing, we are unaware of studies that have explored the adolescent testing experience from multiple perspectives. Social relationships play a critical role in adolescents’ beliefs and behaviors, as they navigate both increased autonomy and ongoing vulnerability [21]. Interactions with parents, HCWs, and peers may have a particularly powerful impact on adolescent HIV testing and linkage to follow-up services. To address this gap in knowledge and guide program improvements, our study aimed to characterize the adolescent HIV testing experience and explain how critical relationships influence adolescent HIV testing and linkage to care or repeat testing. We triangulated perspectives from adolescents, caregivers, and healthcare workers directly involved in adolescent testing.

## Methods

### Study design and population

The current qualitative study was conducted at a national teaching and referral hospital in Nairobi, Kenya. Purposive sampling was used to recruit participants at the voluntary counseling and testing (VCT) clinics, the HIV comprehensive care clinic (CCC), and the Youth Center (YC). Typically, the VCT serves older, unaccompanied adolescents who self-refer for HIV testing, the YC serves accompanied adolescents who present for testing and/or other services, and CCC provides confirmatory testing and enrolment in HIV care. Semi-structured interviews were conducted with eight HIV-positive and eight HIV-negative adolescents aged 13–18 years who had tested for HIV. We focused on adolescents aged 13–18 years because of HCW uncertainty on how to navigate consent, disclosure, and counseling on sexual behavior topics with this age group, as well as considerations of feasibility identifying young adolescents who had been disclosed to and who were able to discuss complex emotional experiences. Recruitment was stratified by HIV status. Four focus group discussions (FGDs) were conducted with caregivers of adolescents who had tested. Caregivers were not necessarily caregivers of adolescent participants. Caregiver FGDs were stratified by adolescent age group (13–15 or 16–18 years) and HIV status to capture a range of perspectives. Two FGDs were held with HCWs who were currently employed in testing adolescents at the VCT and YC. Characteristics of study participants are described in Table 1.

**Table 1:** Sociodemographic characteristics of study participants.

Characteristic	Population		
	Adolescents, *N* = 16	Caregivers, *N* = 22	Healthcare providers, *N* = 25
	Median (IQR) or *n* (%)		
Female	6 (38)	16 (73)	17 (68)
Age	16 (15–17)	42 (38–44)	37 (34–53)
Adolescents aged 13–15 years in household^a^	–	13 (60)	–
Adolescent HIV-positive^b^	8 (50)	13 (59)	–
Education			
Primary	3 (19)	8 (36)	0
Secondary	13 (81)	7 (32)	0
College/polytechnic	0	7 (32)	25 (100)
Years of education	–	12 (8–16)	17 (15–18)
Primary caregiver(s)			
Mom	4 (25)	–	–
Dad	2 (12)	–	–
Both parents	10 (63)	–	–
HIV tests completed			
One	7 (44)	–	–
Two	7 (44)	–	–
Three	2 (12)	–	–
Partner status^c^			
No partner	10 (77)	2 (9)	–
Boyfriend/girlfriend	3 (23)	0	–
Married	0	14 (64)	–
Divorced/separated/widowed	0	6 (27)	–
Employment status			
Professional	–	7 (32)	–
Casual	–	4 (18)	–
Unemployed	–	6 (27)	–
Other^d^	–	5 (23)	–
Employment site or function^e^			
VCT	–	–	6 (26)
Youth Center	–	–	7 (30)
PITC^f^			10 (44)
Years in current clinic	–	–	5 (1–9)
Years of HIV counseling^g^	–	–	10 (6–14)

^a^This refers to the ages of the caregivers’ adolescent children. All other caregivers have adolescents aged 16–18 years.

^b^For caregivers, this refers to the HIV status of their child.

^c^Three adolescents missing partner status information.

^d^Other employment included maize vendor, charcoal vendor, and other vendor.

^e^Two healthcare providers missing information on employment site; PITC, provider initiated testing and counseling; VCT, voluntary counseling and testing.

^f^Provider initiated testing and counseling is an approach to HIV counseling and testing provided by HCW in different places in the hospital, including in-patient or out-patient services.

^g^One healthcare provider missing information on years of HIV counseling.

### Ethical considerations

The current study was reviewed and approved by the University of Washington Institutional Review Board (Protocol #48627) and the Kenyatta National Hospital/University of Nairobi Ethical Review Committee (Protocol #P281/05/2015). All adult participants provided written informed consent, whereas those aged 13–17 years provided written assent and a caregiver provided written consent.

### Data collection

Semi-structured interviews and FGDs were conducted between January and May 2016. Interviews with adolescents focused on barriers and facilitators to HIV testing, involvement of caregivers and peers in test decision-making and testing process, and what the adolescents liked and did not like about their testing experience. FGDs with HCWs and caregivers discussed current HIV testing and disclosure guidelines and practices, perceived challenges to testing, involvement of family and/or peers, and recommendations to improve adolescent testing and linkage. Interviews and FGDs were conducted in Kiswahili or English, digitally recorded, translated (if applicable), and transcribed.

## Analysis

We performed a descriptive analysis [22] using a modified version of the constant comparative approach [23,24] and thematic network analysis [25] to identify key concepts and themes arising within and between participant groups. ATLAS.ti v.7 software (Scientific Software Development GmbH, Berlin, Germany) was used to support coding, data management, and analysis. An initial codebook was created by primary coders based on a subset of transcripts and a literature review. As these codes were applied to more transcripts, additional codes emerged as well as more nuanced themes which led to a further expansion of the codebook. Using the final codebook, half of the transcripts were independently coded by one team member and half by another. Transcripts were exchanged and team members reviewed the coding applied. To identify additional themes, the study team compared coded text within and across HCW, caregiver, and adolescent groups. Major themes were then discussed amongst the larger study group and agreement reached on interrelationships as the organizing theme of this article.

## Results

**Fig. 1:**
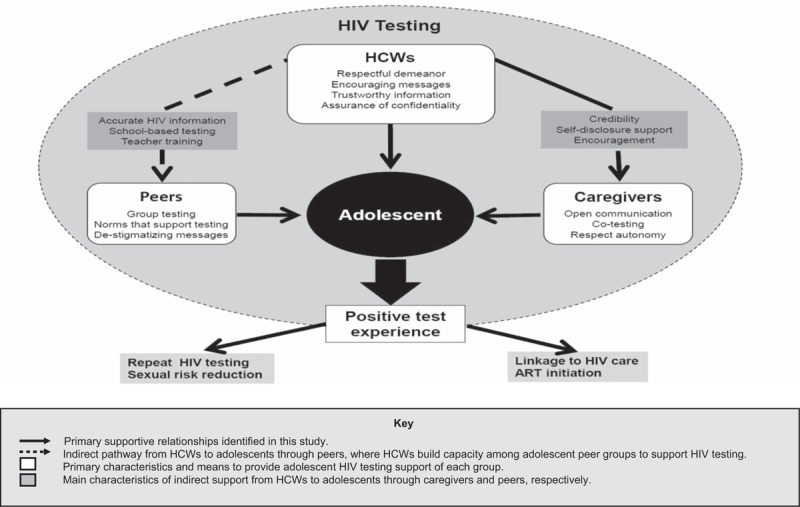
Adolescent HIV testing and support conceptual diagram.

Supportive relationships between adolescents and HCWs and/or adolescents and caregivers played a critical role in whether adolescents were tested and either linked to care or were willing to be tested again in the future. Adolescent and caregiver perspectives on testing did not differ substantially by HIV status, except that some HIV-positive caregivers mentioned reluctance to encourage adolescent testing until caregivers themselves were ready to disclose their own status. HCWs were pivotal in influencing whether adolescents tested and engaged in follow-up services, whereas caregivers’ influence was greatest in pre and posttesting. Peer influence was present, but less critical to testing success because few adolescents disclosed testing or test results with peers. Key features of supportive interactions between adolescents, HCWs, caregivers, and peers are illustrated in a conceptual diagram (Fig. 1). Quotations illustrating supportive interactions are presented in the supplementary appendices, Supplement Table 1, http://links.lww.com/QAD/B94.

The conceptual diagram presents how HCWs, caregivers, and peers in this study support the adolescent HIV testing experience. A positive testing experience was viewed as motivating HIV-positive adolescents to enroll in HIV care and initiate ART, and motivating HIV-negative adolescents to return for HIV testing and practice risk reduction. The words in boxes correspond to the categories of the key themes presented by each group in Supplement Table 1, http://links.lww.com/QAD/B94. ART, antiretroviral therapy.

### HCWs provide critical support during the test experience

There was universal agreement that HCW interactions with adolescents had the largest influence on overall perceptions of the test experience by adolescents. If the experience was positive, HIV-negative adolescents reported increased motivation to promote testing to peers and return for repeat testing, whereas HIV-positive adolescents reported increased motivation to link to treatment and care services.

Although a few adolescents were tested as part of care for another illness through provider-initiated testing and counseling, most adolescents tested because they wanted to know their HIV status. They wanted HCWs to show respect for their concerns and preferences, and not treat them like children. HCWs could demonstrate respect for adolescents by acting friendly, avoiding comments and actions that could be interpreted as rude or ‘harsh’, and acting engaged rather than distracted or dismissive.

‘The healthcare worker should be like a normal human being but she should be humble to the person and respect[ful], even if the person is smaller or underage …’ P12: 18 year old HIV-positive male

Adolescents were concerned about being judged and blamed by HCWs for being sexually active or engaging in risky sex, and exposing themselves to HIV.

‘[T]hat is why I had even refused [to start HIV treatment] … [The counselors] were imagining it was my fault that I am the way I am … they were judging me … they were saying, “Mama, be on the lookout for this girl.”’ P16: 17 year old HIV-positive female

Although HCWs recognized it was important to discuss sexual initiation and risk reduction with adolescents nonjudgmentally, some acknowledged that their personal beliefs, reflecting social norms that adolescents should not be having sex, could negatively influence interactions with adolescents.

‘Sometimes us providers [are accused of] being judgmental … the provider asks questions without knowing the youth is interpreting the question in a different way, so they share information like “That provider was so judgmental, don’t go there” …’ HCW, VCT

The content of HCWs’ messages during pretest counseling was also important. Adolescents wanted encouraging and honest information to prepare them for receiving test results, regardless of the outcome. They appreciated when the HCW would say that *having HIV is not the end of the world.*

‘Before we were tested, she explained to us the importance of getting tested and when you are diagnosed with it, the way you can live … the more she continued talking, the fear also disappeared and that's why I agreed to be tested.’ P6: 17 year old HIV-negative male

In posttest counseling, HIV-positive adolescents felt that a HCW's supportive messages and a clear follow-up plan were especially helpful in building their confidence, resilience, and motivation to enroll in HIV care.

‘She told me that there are many doctors, many engineers, many big people that are HIV-positive, but you will never know, so she encouraged me.’ P5: 16 year old HIV-positive female

‘If the person goes to the clinic and is diagnosed to be positive, the healthcare provider should help him or her how he or she is going to go the hospital, so that the person can be able to get drugs because if you leave him or her that way, he or she might say, “How am going to go to that hospital and start the drugs?” … If the healthcare worker leaves him or her, he or she will go home and will never come back and just wait to die.’ PD 16: 17 year old HIV-positive female

Adolescents appreciated when HCWs gave them some autonomy over the testing process, including options to test alone and have control over results disclosure, and described frustrating situations when their autonomy was not respected.

‘[T]he doctor … wanted to chase me away so that I can come back with my parent, but I told him that this is my status and not my parent's, therefore, if you don’t want to carry out the test, I will go and get tested elsewhere.’ P12: 18 year old HIV-positive male

Although generally supportive of adolescent autonomy, HCWs expressed challenges balancing adolescent preferences with current consent guidelines in Kenya [26]. Unaccompanied adolescents who did not qualify as ‘mature minors’ under Kenyan law (i.e., has child, pregnant, or married) created a dilemma for HCWs who wanted to offer them testing yet feared negative legal repercussions if caregivers were not involved (supplementary appendices, Supplement Table 2, http://links.lww.com/QAD/B94).

‘If you are going to test the adolescent and the parent is not aware, then the legal aspect is a big challenge … [also] you don’t know the reaction you are going to get because the youth, they have suicidal ideas, so [a positive result] can also cause them to commit suicide …’ HCW, YC

## Caregivers can support adolescents by role modeling and respecting autonomy

Most adolescents turned to caregivers for motivation to test and courage to receive the result. Although adolescents were concerned about being judged, they wanted their caregivers to talk to them about testing. Adolescents also wanted caregivers to respect their autonomy about when to test, and support decisions to test once they were ready.

‘HIV testing should never be forced by a parent or other … it's just something you personally should do, not just your mother telling you, “Tomorrow I will be taking [you] for a HIV test” …’ P19: 15 year old HIV-negative male

Several adolescents noted that having a parent get tested for HIV alongside the adolescent, referred to as cotesting, helped reduce fear and motivate engagement in HIV care or prevention.

‘I was afraid but I didn’t show it. So [my mom] was tested first, then I felt “This thing is not bad.”’ P8: 16 year old HIV-negative female

Caregivers universally supported adolescent HIV testing services and felt that it was their responsibility to serve as role models for HIV testing, risk reduction, and ‘positive living.’

‘It's very good for these teenagers to get tested … when he/she reaches that age, if you do not teach him/her about HIV and its risks, and the future life, within no time you will find that the child is already involved in that risk.’ Caregiver of an HIV-negative 13–15 year old

Similar to adolescent wishes, some caregivers also supported cotesting, describing testing together and self-disclosure as a means to demonstrate support.

‘When you accompany this child, that means you should play as a role model, so when you get into the room, you must be ready to be tested together with him/her … [it] is a way of supporting him/her morally or emotionally, and the child will have the confidence, “Now that my parent has been tested,” if he or she is diagnosed with it, “we are here together, at least we are sailing on the same boat.” It's good that you should be tested together.’ Caregiver of an HIV-negative 13–15 year old

Most caregivers wanted to know adolescent test results, regardless of the adolescents’ desire to share them. However, a few caregivers believed it was the right of the adolescent to decide whether to disclose to a parent. Caregivers supporting adolescent autonomy for disclosure still wanted to ensure that a trusted family member or friend was present to offer support to younger adolescents if they received a positive test result.

‘For young adolescents, it's important to be accompanied by the parents/guardian so that you can follow the issues of adherence on drugs and counsel them on the issue of going to the clinic, but the ones who have reached the age of 19, 20, most of them don’t want interference, they feel that you are micro managing him/her … The child should be accompanied [by] the parents if they are below 15 years, and those above that age should be given their space …’ Caregiver of HIV-positive 13–15 year olds

Caregivers, adolescents, and HCWs also noted that a positive relationship between caregivers and HCWs reinforced a positive testing experience. Caregivers noted that HCWs provided support by acting as an independent and credible source of information, helping to reinforce messages about risk reduction or positive living after an HIV diagnosis, and sharing techniques for communicating effectively with their adolescents. Adolescents agreed that HCWs played an important role in educating caregivers on how to support them through the testing experience.

‘If the patient is positive, now you call in the parent, talk through both the parent and the patient, give them some ideas on how to motivate the patient. If HIV-positive, the provider should help the parent counsel the patient …’ P19: 18 year old HIV-negative male

Although HCWs had a major influence over adolescents during testing, they depended on caregivers to support adolescents beyond the clinic to cope with a positive diagnosis and adhere to antiretroviral therapy or to help prevent HIV infection.

‘We actually depend [on caregivers] because they offer very big support, we cannot achieve without them, because you find that the social support system begins with them …. As a provider I finish with you today and they have to walk with other people.’ HCW, YC

## Peer support of HIV testing was limited by stigma and misinformation

Adolescents’ friends and peer groups at school and in their communities played a less significant role in the testing experience compared with HCWs and caregivers. However, peers had the greatest influence over adolescents’ general beliefs about HIV and motivations to test. Although schools provided basic education about HIV, misinformation and HIV stigma were common.

Few adolescents described sharing personal experiences with peers about testing. Adolescents feared being stigmatized and losing friends if HIV-positive or being judged for participating in sexual risk behaviors. Peers could further discourage testing by spreading misinformation about testing or the test facility.

‘I don’t feel that it's ok to go [get tested] with a friend … because they might go in together and maybe this friend doesn’t have HIV but the person has, so he might go and tell his friends out there that this person has HIV, you should not be friends with him.’ P3: 17 year old HIV-negative male

Despite reservations about engaging peers in HIV testing, most adolescents agreed that trusted friends could be an important source of support. Adolescents who had positive testing experiences and had accepted their HIV status were viewed as potential change agents who could spread testing acceptance among their peer network.

‘Instead of gossiping … you can say “Let's talk about HIV and ways we can protect ourselves and how we can help those who are infected by HIV … today is world AIDS day and we talk about how we can help each other, how we can communicate with them so that they can take their drugs well, so that they can be strong, so that they can live long just like you …”’ P9: 16 year old HIV-positive female

Some adolescents mentioned that they could create positive peer pressure to test by testing in groups or accompanying friends for testing, though typically they would not disclose their results. HCWs echoed the views of adolescents that group testing was an important way for adolescents to support each other.

‘Sometimes [adolescents] say … “Please let's go and you accompany me as I test, I fear getting pricked, fear seeing blood, I fear sitting with a stranger” so they just come for support and … they are free talking to you when they are together … and you empower and encourage them to teach each other to lower the chances of getting infected.’ HCW, VCT

HCWs thought they could help mobilize peer support for testing by offering HIV testing and referrals at schools and providing accurate information to reduce stigma.

‘Let's take the services to them, the time we are sitting waiting for them, that's the time they are getting infected …’ HCW, VCT

## Discussion

The current study extends understanding of the importance of social support in HIV prevention and care for adolescents [10,27–29], particularly emotional and informational support [30], by revealing the dynamic interactions among HCWs, caregivers, and peers that influence adolescent HIV testing experiences. Peers could either reinforce or undermine the HCWs’ positive role, which is consistent with prior studies on the mixed role of peers in adolescent risk behaviors [31–33]. These findings support an ecodevelopmental model [34], which posits that dynamic social interactions between the adolescent, family, and peers, across developmental stages and contexts, are important determinants of adolescent risk behaviors.

The prominent role of HCWs has been noted in previous studies on adolescent satisfaction with healthcare and use of HIV services [35]. A HCW's friendly, nonjudgmental, trustworthy, and confidential interaction may improve satisfaction with care and motivation to engage in health services. Although the impact of ‘youth friendly services’ on adolescent HIV testing and care in Kenya is yet to be determined [14,36], our study identified key characteristics of the HCW-adolescent relationship that may be necessary to achieve youth friendly goals.

Adolescent autonomy emerged as an important theme that complicated supportive relationships. Although adolescents wanted support from HCWs and caregivers during testing, they also wanted control over when they tested, with whom, and how they shared their results, illustrating the tension between support and autonomy. This struggle between support and autonomy is a ubiquitous feature of adolescent development, characterized by increasing independence yet ongoing vulnerability to risky behaviors and relationships [21,37]. This study highlights the need for clearer adolescent HIV testing guidelines that describe alternative ways to respect adolescent autonomy within the bounds of current laws.

All groups identified the potential to mobilize peers and offer testing in schools as a way to increase demand for adolescent HIV testing and combat HIV stigma. HCWs could strengthen peer support to adolescents during testing (Fig. 1) by taking services to them and increasing positive norms about HIV testing and acceptance of results. Although schools are promising venues to offer HIV testing and education [38,39], more research is needed to identify optimal roles of peers and schools for adolescent testing and linkage to services [5,18,40–42].

Strengths of this study include the triangulation of both HIV-positive and HIV-negative adolescents, HCW, and caregiver perspectives to generate a more comprehensive understanding of HIV testing experiences. We targeted younger adolescents (≤18) because this group is underrepresented in HIV research [43]. As this study was conducted in a setting with extensive experience in HIV testing and care, participants’ testing experiences may differ from other facilities or regions. In addition, our sample of adolescents was too small to fully explore differences in testing experiences between subgroups (e.g., age, sex, romantic partnerships, and testing venue) [10,11].

## Conclusion

Supportive interactions that overcome fears of testing and a positive result are important to improve adolescent HIV testing experiences and follow-up services. Results have informed development of an ongoing clinical training intervention to improve adolescent engagement in HIV care in Kenya. Cotesting, clear consent guidelines, and provider and caregiver trainings on support and messaging may help to accelerate progress toward achieving the ‘90-90-90’ targets for this key population.

## Acknowledgements

We would like to thank all participants for making this study possible and the research and clinic staff at the University of Nairobi and Kenyatta National Hospital who supported data collection, transcription, and interpretation.

The study was supported by National Institutes of Health (NIH) implementation science supplement to the University of Washington Center for AIDS Research (CFAR), a NIH funded program (P30 AI027757). CFAR is supported by the following NIH Institutes and Centers: NIAID, NCI, NIMH, NIDA, NICHD, NHLBI, and NIA.

Contributions: P.K.K., J.A.S., G.O., D.W., A.D.W., H.M., C.M., P.M.M., D.B., G.C.J.-S., K.S.W., and K.M.B.-S. conceptualized the study design. A.D.W., H.M., P.K.K., J.A.S., D.W., and G.C.J.-S. developed the study materials and H.M. conducted the interviews and focus groups. K.S.W. and K.M.B.-S. conducted the analysis with input from H.M., A.D.W., P.K.K., J.A.S., G.C.J.-S., and G.O., K.S.W., K.M.B.-S. drafted the article and all authors contributed to the final article.

### Conflicts of interest

There are no conflicts of interest.
